# The Relationship between Physical Activity and Quality of Life in Postmenopausal Women: A Cross-Sectional Study

**DOI:** 10.3390/healthcare12191963

**Published:** 2024-10-01

**Authors:** Maria Tsekoura, Zacharias Dimitriadis, Andreas Gridelas, Argiro Sakellaropoulou, Georgios Kolokithas

**Affiliations:** 1 Laboratory of Clinical Physiotherapy and Research, Department of Physiotherapy, University of Patras, 26504 Rio, Greece; 2 Department of Physiotherapy, University of Thessaly, 35132 Lamia, Greece; 3 Elderly Open Care Centers Patras, 26226 Patras, Greece

**Keywords:** postmenopausal women, physical activity, quality of life

## Abstract

Background: Postmenopausal women frequently encounter a range of symptoms, including fatigue, diminished physical strength, reduced energy levels, vasomotor symptoms such as hot flushes, and vaginal atrophy, all of which adversely affect their overall quality of life. Engaging in physical activity and structured exercise may effectively alleviate these symptoms and enhance overall well-being. The present study aimed to investigate the relationship between physical activity and quality of life in postmenopausal Greek women. Methods: This cross-sectional clinical study included 219 postmenopausal women. Women with natural menopause for at least 12 consecutive months were enrolled in this descriptive, cross-sectional study. The female participants were asked to fill out the International Physical Activity Questionnaire-short form (IPAQ), the Hospital Anxiety and Depression Scale (HADS), and the EuroQol (EQ-5D-5L) instrument. Anthropometric measurements included weight, height, and waist circumference measurements. Results: A total of 219 postmenopausal women with an age of 61.4 ± 6.1 years and body mass index (BMI) of 25.6 ± 3.7 kg/m^2^ were studied. Out of the total postmenopausal women studied, 64.8% were physically active. The mean value of MET-min/week was M = 1383.46 ± 1030.12. Physical activity among postmenopausal Greek women showed a strong correlation of PA with quality of life (r = 0.5; *p* ≤ 0.001) and age (r = 0.55; *p* ≤ 0.001) and a medium correlation with the HADS (r = 0.4; *p* ≤ 0.05). Conclusions: There was a 64.8% prevalence of physically active postmenopausal Greek women. The findings underscore the significance of fostering physical activity and quality of life among postmenopausal women to formulate efficacious therapeutic interventions. The results demonstrate a correlation between physical activity and the age of female participants, quality of life, and the HADS and can be used to improve postmenopausal women’s physical activity levels, which is recommended as a strategy for improving the quality of life in postmenopausal women.

## 1. Introduction

Menopause, defined as the cessation of menstruation for 12 months [1,2,3], is a normal physiological change occurring in middle age in a woman’s life [3]. It usually affects women between the ages of 46 and 52 and signifies the aging of a woman’s reproductive system [2]. Menopause transition brings in significant physiological changes in the bodies of women (e.g., vasomotor and sleep disturbances, vaginal dryness, pain, and fatigue), affecting well-being and quality of life (QoL) [2,4,5]. Mental symptoms such as irritability, anxiety, and feelings of depression also increase around menopause [6,7]. Postmenopausal symptoms are primarily attributed to the decreased levels of circulating estrogen [8,9,10,11]. Menopause is also associated with significant changes in body composition, including increases in fat mass and peri-abdominal and visceral fat [12,13].

Taking into account that women spend one-third of their lives in the postmenopausal stage, it seems important for health professionals to design preventive strategies. Overall, a multifaceted approach that includes lifestyle modifications, nutritional support, exercise, and the cautious use of hormone therapy is essential for optimizing health in postmenopausal women [13,14]. It is well established that regular physical activity (PA) provides health benefits [15]. PA is a behaviour involving human movement that provides physiological benefits, including increased energy expenditure and improved physical fitness [3,16]. PA in postmenopausal women may improve cardiometabolic, physical, and psychosocial health, enhancing their quality of life by mitigating detrimental changes in health during and after menopause [17,18].

PA seems beneficial [19,20,21,22], but evidence provides mixed results. In a study conducted by Baral et al. [23], the results show that physical activity is one of the critical predictors of the quality of life among menopausal women. In addition, de Azevedo et al. record that women who maintained their total habitual physical activity at more than 60 min per day had reduced menopausal symptoms and improved quality of life [19]. However, some studies have recorded inconsistent results [24,25]. In addition, the results of the systematic review show that data regarding exercise for vasomotor menopausal symptoms are not enough [17].

Regarding mental issues, evidence suggests that engaging in PA protects against anxiety symptoms and depression. Understanding the relationship between PA and postmenopausal depression can help health professionals in developing better screening, management, prevention, and intervention strategies for postmenopausal women [26,27,28].

Studying the correlations between PA and QoL may help health professionals such as doctors, nurses, and physiotherapists design health-promoting strategies and effective interventions to improve the QoL and well-being of postmenopausal women. The aging rate of the Greek population is among the fastest compared to all other European Union countries [29]. Given the rapidly aging nature of our society and the fact that the aging process may be affected by genetics and environmental factors [29], it seems important to investigate the physical activity behaviours of different populations. Notwithstanding the established correlation between physical activity and quality of life in postmenopausal women, as evidenced by contemporary research [30,31], there exists a notable deficiency of data pertaining specifically to postmenopausal Greek women. Thus, this study aims to assess the relationship between physical activity levels and quality of life and other variables (e.g., anxiety and depression) in postmenopausal Greek women.

## 2. Materials and Methods

This is a cross-sectional analytical study involving 219 older adults living in western Greece. This study was conducted from November 2022 to April 2023. The Ethics Committee of the University of Patras, Greece, approved the study protocol (number 6238/2022).

### 2.1. Participants

The participants in this cross-sectional study were recruited from the 2nd Open Care Centre of Patras for the Elderly in Patras city and from the University Hospital of Patras. Female participants were recruited using flyers, posters, and advertisements. Prior to recruitment, participants were screened for eligibility. Eligible participants had to be 1. women currently living in Greece and 2. women with natural menopause for at least 12 consecutive months. Women who were unable and/or unwilling to answer were excluded from this study. All women signed an informed consent form prior to their inclusion. All participants lived in Achaia County.

Sample size calculation was performed with the G* power 3.1 software. Based on the primary hypothesis of this study, the sample size calculation revealed that for detecting a significant correlation of moderate effect size (r = 0.3), and by using an α = 0.05, a statistical power of 80%, and a two-tailed hypothesis, the minimal required sample size was 84 participants.

### 2.2. Data Collection Procedure

The data were collected through a face-to-face interview. A structured interview schedule was used to collect information on the participants’ age, general health status, education level, time since last menstruation, hormonal therapy use, socioeconomic status, and smoking habits. Menopausal status was determined based on a self-report. Osteoporosis diagnosis was confirmed by participants’ recent dual-energy X-ray absorptiometry (DXA) measurement.

### 2.3. Outcome Measures

Physical Activity assessment: The Greek version of the International Physical Activity Questionnaire-short form (IPAQ) was used. This tool assesses PA levels and has acceptable measurement properties [32,33]. This questionnaire provides information about the time spent involved in three PA intensity levels: (a) walking, (b) moderate, and (c) vigorous. Women were divided into inactive (low PA level) and active (moderate and high PA levels) based on duration, days of exercising, and the metabolic equivalent of tasks (METs) [32,33]. The levels of PA [high (>3000 MET-min/week), moderate (600–3000 MET-min/week), and low (<600 MET-min/week)] were established based on the American Heart Association’s recommendations on minimum weekly PA for adults (Physical Activity Guidelines) [34].

Anxiety and Depression assessment: The Greek version of the Hospital Anxiety and Depression Scale (HADS) was used [35]. This tool is a widely utilized tool for assessing anxiety and depression in patients with various medical conditions. Several studies have explored its effectiveness and psychometric properties, highlighting its relevance in clinical settings. The term ‘hospital’ in its title suggests that it is acceptable in such a setting, and many studies conducted worldwide confirmed that it is valid when used in community settings and primary care medical practice [36]. The Greek version of HADS shows good psychometric properties [35,37]. The HADS comprises 14 questions and takes 2–5 min to complete [38]. It consists of two subscales: the anxiety subscale (HADS-A) and the depression subscale (HADS-D). The cut-off scores for the total score and subscales are as follows: (a) 0–7 normal, (b) 8–10 mild/doubtful, and (c) >10 moderate/severe (clinical anxiety or depression) [38].

Quality of Life assessment: QoL was assessed via the Greek version of the five-level EQ-5D questionnaire (EQ-5D-5L). This instrument evaluates five health domains: mobility, self-care, usual activities, pain/discomfort, and anxiety/depression [39]. The answers given to ED-5D can be converted into an EQ-5D index, with utility scores anchored at 0 for death and 1 for perfect health. The EQ-5D also includes a Visual Analogue Scale (EQ-VAS) aiming to capture respondents’ ratings of their ‘health today’ on a scale from 0 to 100 [39]. The EQ-5D is valid and reliable for various diseases [40,41].

### 2.4. Anthropometric Measurements

Anthropometric assessments for each participant were conducted by a highly skilled physiotherapist in the morning following a fasting period of no less than eight hours. Height was measured to the nearest 0.5 centimetres, while weight was recorded to the nearest 0.1 kg. Body mass index (BMI) was calculated as the quotient of body mass (in kilograms) divided by the square of stature (in metres) [42].

### 2.5. Waist Circumference Measurement

Waist circumference, quantified in centimetres (cm), was assessed utilizing a flexible, non-elastic plastic tape measure positioned at the horizontal plane located equidistantly between the inferior margin of the lowest rib and the iliac crest. Measurements were taken to the nearest 0.5 cm subsequent to a normal expiration and were conducted twice at each designated site. In instances of any discrepancies between the two measurements, the greater value was documented [43].

### 2.6. Data Analysis

SPSS 28.0 was used for the statistical analysis. The normality of the distribution of quantitative variables was tested with the Kolmogorov–Smirnov test. The descriptive characteristics were presented as the means and standard deviations for quantitative variables and as the means of frequency for qualitative variables. Spearman’s correlation coefficient matrix for the IPAQ and all variables was performed. Categorization was made according to Cohen (r = 0.10 small, r = 0.30 medium, and r = 0.50 large) [44]. The significance level was set at *p* ≤ 0.05 for all tests.

## 3. Results

### 3.1. Participants’ Characteristics

The sample consisted of 219 Greek women aged between 54 years and 78 years, with a mean age of 61.4 ± 6.1 years. The mean age at menopause is 47.31 ± 5.46 years. The majority of women were married (63.3%) and retired (66.2%). Participants’ characteristics are presented in Table 1.

The average BMI was 25.6 ± 3.7, and the waist circumference was 101 ± 8.7 cm. A total of 39.7% (*n* = 87) of the female Greek participants were diagnosed with osteoporosis.

### 3.2. Physical Activity Participation

The results show that 37.4% of all female participants exhibited low physical activity levels, 45.2% exhibited moderate physical activity levels, and 20% exhibited high physical activity levels. The PA level of the female participants ranged from 500 to 3358 MET-min/week. The majority of women (45.6%) showed a moderate (600–3000 MET-min/week) or high (>3000 MET-min/week) level of PA (19.1%). In 35.1% of women, PA was insufficient (<600 MET-min/week).

The findings of this study demonstrated that PA was strongly correlated with age (r = 0.55; *p* ≤ 0.001) and QoL (r = 0.5; *p* ≤ 0.00) and moderately correlated with the total HADS score (r = 0.45; *p* ≤ 0.001). PA showed a medium to low relationship with anxiety (r = 0.39; *p* = 0.02). All other variables had low correlations with the IPAQ score. Table 2 presents the correlations between the IPAQ and all other variables.

## 4. Discussion

To our knowledge, this is the first study to examine PA and QoL in postmenopausal Greek women. The key findings from this research demonstrated a clear relationship between PA and QoL among female participants. This result provides important health-related data of a group not previously studied in Greece.

Overall, our results show that 35.1% of postmenopausal women were inactive. These results are in accordance with other studies [3,45,46]. Physical inactivity places women’s health at risk during menopause (e.g., it reduces the risk for cardiovascular health complications and improves bone health and muscle mass and function) but also increases menopausal problems [14]. Therefore, PA may be an essential strategy to consider when addressing the changes caused by menopause.

The results of the present study show a strong relationship between PA and the ages of the participants. Age-related decline in PA and functional fitness is observed in older women, impacting muscle strength, flexibility, and endurance due to the natural aging process [47]. The World Health Organization recommends 150 to 300 min of weekly moderate-intensity exercise [48]. Considering the benefits of PA on the health and well-being of women as they age, health professionals should encourage women to stay active and promote exercise programs to improve their quality of life [49]. It seems important to understand the factors that influence older adults’ PA engagement to develop strategies to improve PA participation among older women [48]. Τhe promotion of physical activity by health professionals is important to encourage more older people to be active in order to develop, maintain, and recover their capacity to receive help [50].

As was expected, PA has a relationship with QoL among postmenopausal Greek women. The relationship between PA and QoL among postmenopausal women is significant, as evidenced by various studies [31,51]. QoL, in general, can refer to the level of satisfaction a person feels with various aspects of their life. Engaging in PA can alleviate menopausal symptoms and enhance overall well-being [40], indicating the importance of promoting PA for healthy aging [31]. Future research should involve the intervention testing of different modes and intensities of PA to precisely characterize the effects of various forms of PA on aspects of QoL and well-being [52].

The results also record a medium relationship between PA and anxiety. In a recent study, researchers indicated that PA levels are related to anxiety and stress in postmenopausal women. Active women with higher perceived social support exhibit lower psychological distress and better mental health [53]. The incidence of anxiety disorder in menopausal women is high, and its severity may be related to the severity of the menopausal syndrome. The term anxiety is also often used to describe symptoms like feeling on edge, worrying, and developing specific fears and may be influenced by environmental and biological factors. It seems important to explore and discuss these issues in future research [54]. Early screening and clinical intervention for the risks identified here could reduce the possibility of developing anxiety symptoms during and post menopause [55]. The results indicate that engaging in regular physical activity can alleviate psychological distress, including anxiety and stress, particularly during the menopausal transition.

Regarding depression, the latest data from a health survey conducted in Greece showed that one out of four postmenopausal women in Greece screened positive for depression [56]. Engagement in PA may help overall health and well-being during aging by reducing depression and menopausal symptoms [57]. However, the literature shows that several risk factors for depression may also be important, such as various lifestyle factors (e.g., smoking), socioeconomic or financial status, the lack of family or social support, medical and health factors (e.g., chronic diseases), and reproductive health factors (e.g., menstrual cycle length) [8,55,56]. Therefore, it is necessary to improve screening and lifestyle modification approaches that could be helpful in reducing depression symptoms.

This study found no significant relationship between PA and waist circumference (medium-to-low correlation was recorded). However, waist circumference is a significant indicator of cardiometabolic risk, with studies showing positive correlations between waist circumference and visceral fat content. These results highlight the importance of accurate waist circumference measurement in assessing health risks [58,59]. In other studies, PA had significant independent effects on weight or waist circumference [60,61]. Waist circumference may increase independently of old age. Women who are postmenopausal tend to have larger waists than women who remain premenopausal or early perimenopausal [62]. It would be interesting to study the response to exercise and physical activity in women who participated in the present study. Future research addressing dietary intake is needed to better determine how weight or waist circumference are influenced.

### 4.1. Clinical Significance

These findings underscore the importance of PA, which is recommended as a strategy for improving the quality of life in postmenopausal women. Therefore, health professionals should design health interventions to reduce sedentary behaviour among older women [38]. As the population of women over 60 grows, it is important to identify strategies to maximize health and minimize disease burden [63]. This study recommends incorporating PA and exercise interventions into postmenopausal care programs. Healthcare professionals could be viable conduits for PA promotion [64]. Regularly assessing a patient’s level of PA can provide valuable insights into her/his health status. It is also an important first step that can lead to important intervention opportunities [65].

### 4.2. Limitations

There are several limitations in this study. The main limitation is that the present study was conducted in one region in western Greece (Achaia), affecting the generalizability of the results. Female participants were recruited from a few centres in this region, which may influence the sample characteristics. Further research should take into account more characteristics of postmenopausal women across a range of socioeconomic settings. In addition, no specific instrument for menopause was used for QoL measurement. Further research is needed with large representative samples of the general older adult population to further elucidate the association between PA and QoL. In the present study, data collection regarding PA was based on the IPAQ (a self-reported questionnaire), which may be subject to recall bias. Future research should also include objective measurements (e.g., accelerometers, pedometers) [66].

## 5. Conclusions

The percentage rate of active postmenopausal Greek women was 64.8%. The results demonstrate a correlation between PA and the age of female participants, QoL, and the HADS. The results highlight the need for health professionals to promote PA among postmenopausal women. More research is needed to clarify the precise association of specific characteristics of older women with PA and other factors.

## Figures and Tables

**Table 1:** Participants’ characteristics.

Variable	Total Participants (*n* = 219)
Number, SD	
Age (years)	61.4 ± 6.1
Menopause age	47.31 ± 5.46
Drug (number)	2.5 ± 1.4
Comorbidities (number)	2.0 ± 1.0
Osteoporosis diagnosis	87 (39.7%)
BMI (kg/h^2^)	25.6 ± 3.7
Waist circumference (cm)	101 ± 8.7
METs	1383.46 ± 1030.12
EQ-5D-5L score	2.8 ± 1
EQ VAS score (%)	71 ± 11
HADS total score HADS anxiety HADS stress	15 ± 3.3 10.1 ± 1.3 9.4 ± 0.3
Number, percentage	
Physical activity Low active Moderate active High active	77 (35.1%) 100 (45.6%) 42 (19.1%)
Educational level High school University Other	120 (54.7%) 76 (34.7%) 23 (10.5%)
Marital status Married Widowed Divorced Unmarried	141 (63.3%) 25 (11.1%) 22 (10%) 31 (14.5%)
Work status Employed Unemployed Retired Student	27 (12.3%) 52 (23.7%) 145 (66.2%) 1 (0.4%)
Comorbidities (the most frequent comorbidities) Hypertension High cholesterol Arthritis Osteoporosis Diabetes Hypothyroidism Respiratory disease Anemia Stroke Kidney disease	80 (36.5%) 79 (36%) 67 (30.5%) 59 (26.9%) 50 (22.83%) 34 (15.5%) 19 (8.6%) 11 (5%) 9 (4.1%) 6 (2.7%)
Currently smoking Yes	23 (10.5%)

BMI: body mass index; METs: Metabolic equivalent of tasks; HADS: Hospital Anxiety and Depression Scale; EQ-5D-5L: Euro quality of life; EQ-VAS: Euro quality of life Visual Analogue Scale.

**Table 2:** Spearman correlation coefficients for physical activity and other factors.

Variable	HADS	EQ-5D-5L	Menopause Years	Age	Anxiety	Stress	Waist CC	Comorbidities	BMI	Drugs
Physical Activity	r = −0.45 *p* = 0.03	r = 0.5 *p* ≤ 0.001	r = 0.21 *p* = 0.14	r = −0.55 *p* ≤ 0.001	r = −0.39 *p* = 0.02	r = −0.20 *p* = 0.04	r = 0.29 *p* = 0.03	r = −0.20 *p* = 0.11	r = 0.15 *p* = 0.13	r = −0.18 *p* = 0.6

BMI: body mass index; HADS: Hospital Anxiety and Depression Scale; EQ-5D-5L: Euro quality of life; Waist CC: waist circumference.

## Data Availability

The data that support the findings of this study are available from the corresponding author upon reasonable request.
